# Effects of Dysbiosis and Dietary Manipulation on the Digestive Microbiota of a Detritivorous Arthropod

**DOI:** 10.3390/microorganisms9010148

**Published:** 2021-01-11

**Authors:** Marius Bredon, Elisabeth Depuydt, Lucas Brisson, Laurent Moulin, Ciriac Charles, Sophie Haenn, Bouziane Moumen, Didier Bouchon

**Affiliations:** 1 UMR CNRS 7267, Ecologie et Biologie des Interactions, Université de Poitiers, F-86073 Poitiers, France; marius.bredon@univ-poitiers.fr (M.B.); elisabethdepuydt@outlook.com (E.D.); lucas.brisson7@gmail.com (L.B.); bouziane.moumen@univ-poitiers.fr (B.M.); 2 Eau de Paris, Direction de la Recherche et du Développement pour la Qualité de l’Eau, R&D Biologie, F-94200 Ivry sur Seine, France; laurent.moulin@eaudeparis.fr (L.M.); ciriac.charles@anses.fr (C.C.); sophie.haenn@eaudeparis.fr (S.H.); 3 Agence Nationale de Sécurité Sanitaire de l’Alimentation, de l’Environnement et du Travail, F-94700 Maisons-Alfort, France

**Keywords:** microbiota, dysbiosis, isopods, metagenomics, lignocellulose

## Abstract

The crucial role of microbes in the evolution, development, health, and ecological interactions of multicellular organisms is now widely recognized in the holobiont concept. However, the structure and stability of microbiota are highly dependent on abiotic and biotic factors, especially in the gut, which can be colonized by transient bacteria depending on the host’s diet. We studied these impacts by manipulating the digestive microbiota of the detritivore *Armadillidium vulgare* and analyzing the consequences on its structure and function. Hosts were exposed to initial starvation and then were fed diets that varied the different components of lignocellulose. A total of 72 digestive microbiota were analyzed according to the type of the diet (standard or enriched in cellulose, lignin, or hemicellulose) and the period following dysbiosis. The results showed that microbiota from the hepatopancreas were very stable and resilient, while the most diverse and labile over time were found in the hindgut. Dysbiosis and selective diets may have affected the host fitness by altering the structure of the microbiota and its predicted functions. Overall, these modifications can therefore have effects not only on the holobiont, but also on the “eco-holobiont” conceptualization of macroorganisms.

## 1. Introduction

Animal holobionts are assemblages of a host and a complex and rich microbiota, resulting in multiple interactions between bionts (i.e., each associated species in the holobiont) [1]. The structure of the microbiota depends on several factors including host diet, host genotype, host immune system, and microbe-microbe interactions [2]. The stability of microbiota helps to prevent the colonization by exogenous bacteria and the adverse consequences that can result in [3]. But this homeostasis is continuously challenged by abiotic (e.g., soil, air, temperature) and biotic factors (e.g., environmental microbes) that can modify and contribute to the dynamics of microbial communities [4,5,6]. This is particularly the case of the digestive microbiota which is composed of autochthonous bacteria, the residents, and allochthonous bacteria, the exogenous ones, which refer to transient food-borne microbiota [7,8]. The balance between resident and transient bacteria can vary from one holobiont to another and in accordance with its life-history traits. For example, the microbiota of animals such as mammals shows a certain stability due to the high proportion of indigenous bacteria, while that of other animals such as terrestrial isopods is highly dynamic due to environmental filtering [9].

It has also been shown that in mammalian hosts, diet and phylogeny both influence bacterial diversity of the digestive tract, which increases from carnivory to omnivory to herbivory [10]. Adaptation to a plant-based diet was an evolutionary breakthrough in mammals, leading to two alternative strategies corresponding generally to foregut fermenters and hindgut fermenters [10]. In dairy cattle, Firmicutes, Bacteroidetes, and Proteobacteria are predominant bacterial phyla and the relative abundances of the genes involved in carbohydrate metabolism were overrepresented in the digesta samples of forestomaches [11].

Compared to mammals, most insect guts contain relatively few microbial species. Some species harbor highly specialized gut bacteria, while others are colonized only by opportunistic environmental bacteria [12]. In contrast to ruminants, which rely solely on gut microorganisms for cellulose digestion, some insects encode cellulases in their own genomes. Termites, one of the most remarkable examples, are capable of degrading lignocellulose by combining their own enzymatic activities with those of their microbiota [13]. On the contrary, microbial symbionts are generally absent or present only in low numbers in caterpillar guts [14]. Isopods are keystone species in the environment because of their contribution to litter degradation and to the enrichment of biofilms that they consume [15,16,17,18]. As detritivores, these crustaceans are highly permeable to environmental bacteria which constitute an important part of their digestive microbiota [19,20,21,22]. They thus interact with many microbes, which makes them interesting holobionts because of multiple interactions that bind their various bionts [9]. They harbor rich and diverse bacterial communities, including the reproductive parasite *Wolbachia* [23], a vertically transmitted sex parasite [24]. Their microbiota is very variable and depends on factors such as the local environment as well as the sex of individuals and tissues [20]. Variations between males and females are all the more important in populations where *Wolbachia* are present: the presence and abundance of other bacteria are affected in *Wolbachia* infected individuals [19,25]. Environmental factors can also modify microbiota in isopods. For example, the increase in temperature leads to the loss of bacteria belonging to *Actinobacteria* in the common rough woodlouse *Porcellio scaber* [26]. In sympatric populations of the marine isopod *Jaera albifrons* the variance in the microbiota composition over a year was mainly explained by the season and to a lesser extent by sex and geographical origin [27]. The composition of the digestive microbiota of *P. scaber* is significantly affected by the host diet in particular by the amount of biofilms it ingests [28].

Microbiota structuration of isopods depends therefore on several biotic, abiotic, and genetic factors. These microbiota are not fixed in time and space because bionts change according to the host and its environment. These changes directly impact the physiological processes in which microbial communities are implicated [25]. Changes in the diet composition, like the proportion of lignocellulose compounds (i.e., cellulose, hemicellulose, and lignin), could therefore have consequences on microbiota structuration. The main component of plants, lignocellulose, is one of the central food sources for terrestrial isopods. Its degradation by isopods is possible thanks to close interactions between the host and its microbiota [21,22]. Variations in the composition or the amount of lignocellulose in the food of isopods could thus modify biont contributions to digestive processes and change the key players.

In this study, we disrupted the digestive microbiota of the common pill-bug *Armadillidium vulgare* by exposing individuals to starvation to induce dysbiosis. Lignocellulose being a major food source for the isopods, we then varied its components to study the consequences on their digestive bacterial communities. Apart from the microbiota of the hindgut, we have also focused on the microbiota of the hepatopancreas because this digestive tissue, although more isolated, may be permeable to certain bacteria that contribute to the digestion of lignocellulose [20,21,29]. Microbial communities in digestive tissues (hepatopancreas (i.e., all the caeca) and hindgut) were analyzed using 16S metagenomics, making it possible to study the variations in the composition of the microbiota as a function of the diet. This analysis allowed us to (i) precisely identify the modifications of the digestive microbiota of *A. vulgare* according to the lignocellulose composition of the diet, (ii) to evaluate the impact of a dysbiosis and diet manipulation on its structure, and (iii) establish the possible consequences for the host.

## 2. Materials and Methods

### 2.1. Biological Model and Experimental Design

**Figure 1:**
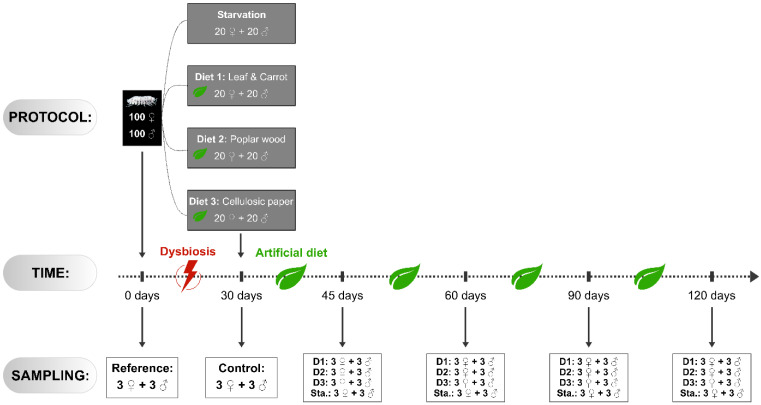
Experimental design. Two hundred individuals were selected and subjected to one month of starvation. Then four batches of 20 males and 20 females were created and subjected to several diets for up to 90 days: Starvation (Sta.), Leaf and Carrot (D1), Poplar wood (D2), and Cellulosic paper (D3). Several individuals were sampled at different times: 0 days (t_0_), 30 days (t_30_), 45 days (t_45_), 60 days (t_60_), 90 days (t_90_), and 120 days (t_120_). Please refer to the Materiel and Methods for more details.

The experiments took place over 120 days. A batch of 100 females and 100 males of the common pill-bug *Armadilidium vulgare* were selected for all experiments. Individuals were all adult siblings from our laboratory, of equal age (1-year-old) and similar weight. They all belonged to the same genetically controlled lineage originating from Helsingör (Helsinor, Denmark). This lineage was selected because it is not infected with *Wolbachia,* to avoid the adverse effects of the presence of this dominant facultative symbiont on the host microbiota. Males and females were separated during all experiments. Before starting experiments, three males and three females were the samples for dissection as described below. These six individuals constituted the reference sample (Figure 1).

To empty the gut content, the pill-bugs were starved for 30 days in plastic boxes on sterile sand at 20 °C under natural photoperiod. Humidity was maintained by a regular sprinkling of double-distilled water to avoid possible contamination by bacteria. Feces and any cadavers were removed daily to limit coprophagia and cannibalism, and to avoid recolonization of the digestive tract by bacteria.

After these 30 days of starvation, pill-bugs were divided into three batches of 40 individuals (20 males and 20 females separately) in boxes on sterile sand. These animals were subjected to three diets with different lignocellulosic compositions (Figure 1): Diet 1 (D1), enriched in cellulose and hemicellulose, was composed of linden leaf and carrots (corresponding to the standard diet provided to pill-bugs bred in our laboratory)—Diet 2 (D2), enriched in lignin, was composed of poplar wood and Diet 3 (D3), enriched in cellulose, was composed of cellulosic paper (Whatman™ 3MM CHR Cellulose). The relative proportions of lignocellulose in the components of these diets were as follows: Linden leaf (40% cellulose, 20% hemicellulose and 20% lignin; [30]), carrots (10% cellulose, 6% hemicellulose and 3% lignin; [31]), Poplar (23% cellulose, 52% hemicellulose and 16% lignin; [32]), cellulosic paper (98% cellulose, Whatman^TM^). All diets were previously sterilized in Stericlin^®^ self-seal pouches (each pouch containing one ration) using high-pressure steam. Finally, another batch of 20 males and 20 females was subjected to continuous starvation (Sta.). Animals were subjected to those conditions for several days, during which 3 males and 3 females were sampled at five different time points (Figure 1): 30 days (t_30_, control samples), 45 days (t_45_), 60 days (t_60_), 90 days (t_90_), and 120 days (t_120_). At each time point, the individuals were weighed and counted.

The European Directive 2010/63/EU and the French decree n°2013-118 regulating animal research do not require an ethical evaluation prior to research on arthropods. However, we complied with the 3Rs ethical rules: even though the replacement was not possible, we minimize the number of animals used for the study. For DNA extraction, animals were killed by freezing before dissection. After the end of the experiment, surviving animals were returned to standard rearing conditions.

### 2.2. DNA Extraction

A total of 108 sampled individuals were dissected to extract their DNA. Prior to dissection, all individuals were surface sterilized using sodium hypochlorite (1%). Tissues were then dissected out using sterilized instruments. All tissues were rinsed in Ringer solution to avoid cross-contamination between tissues. Caeca and hindguts (with their contents) from 3 males and 3 females were kept as separate samples, and the remaining tissues were discarded. Each pooled sample was frozen in liquid nitrogen and ground with a mortar and pestle. The resulting powders were processed using a DNA/RNA extraction kit (Qiagen, Courtaboeuf, France) to extract DNA according to the manufacturer’s protocol. The extracted DNA was stored at –20 °C until use.

### 2.3. Sequencing

The taxonomic profile of samples was identified through 16S metagenomics sequencing. For each diet and time point, equimolar amounts of amplified DNA from the 3 biological replicates of the same tissue and sex were pooled, resulting in 72 samples (Supplementary Materials Additional file 1). V4 regions of the bacterial 16S rDNA genes were amplified by PCR using the universal primers 515F (GTG CCA GCM GCC GCG GTA A) and Y-806RB (GGA CTA CNV GGG TWT CTA AT) [33] with Illumina index adapters. PCR was performed as recommended by Pichler et al. [34]: 3 min at 95 °C followed by 30 cycles of 30 s at 95 °C, 30 s at 56 °C, and 1 min at 72 °C and a final elongation step at 72 °C for 5 min. The PCR products were confirmed by gel electrophoresis and DNA was quantified using a Qubit 2.0 Fluorometer (Invitrogen, Villebon sur Yvette, France). Then samples were purified according to the Illumina protocol [35] with AMPure XP (Beckman Coulter^TM^). PCR was performed as follow to add an index to amplified samples: 3 min at 94 °C followed by 12 cycles of 15 s at 94 °C, 30 s at 57 °C, and 30 s at 68 °C, and finally 5 min at 68 °C. Samples were then purified as described above, and amplicon length was controlled on a DNA chip (Bioanalyzer 2100, kit DNA 1000). Finally, the 72 resulted in metagenomic libraries were sequenced on an Illumina MiSeq at the laboratory of “Eau de Paris” (www.eaudeparis.fr), generating 2 × 250 bp paired-end reads.

### 2.4. Metagenomic Data Analysis

Reads were processed through Qiime2 (version 2019.7; [36]): low-quality reads and sequencing adaptors were removed using Cutadapt [37], and sequencing errors were corrected with Dada2 [38] using custom parameters (--p-trunc-len-f 150 --p-trunc-len-r 160 --p-trim-left-f 40 --p-trim-left-r 40). Remaining reads with > 99% of similarity were clustered with VSEARCH [39] and they were classified by taxon using a taxonomic database based on Sklearn [40]. Results were deep analyzed with the Phyloseq package [41] in R (version 3.6.1; [42]) as for the analysis of taxonomic diversity. Differential relative abundance analysis of microbiota was carried out using DESeq2 (version 1.24; [43]). For this analysis, males and females of the same condition and same time step were pooled because the sex of the host had no significant effect on microbial communities (see below). PICRUSt2 [44] was used to predict functions in samples and results were then compared using the nonparametric Kruskal–Wallis rank-sum test. Non-metric multidimensional scaling (NMDS) and principal coordinate analysis (PCoA) were carried out with Vegan package [45] on the Bray–Curtis dissimilarity matrices constructed from the abundance of taxa in samples and the abundance of predicted functions respectively. Diversity indexes were calculated with Qiime2. Finally, all statistical analyses were performed using R software (version 3.6.1) and figures plotted using the ggplot2 package [46].

## 3. Results

Two hundred individuals of *A. vulgare* were subjected to one month of starvation (t_0_–t_30_, Figure 1) to disrupt the microbiota and lead to dysbiosis. During that period, a total of 10 females and 1 male died. There was no significant difference in weight before and after the month of starvation in males (154 ± 25 mg on average at t_0_ and 152 ± 24 mg on average at t_30_; t-test: t = 0.65, df = 98, *p* = 0.52) and in females (192 ± 39 mg on average at t_0_and 184 ± 40 mg on average at t_30_; t-test: t = 1.46, df = 89, *p* = 0.15). The remaining animals were then fed with several diets with different lignocellulosic compositions to evaluate their impact on the composition of digestive microbiota in caeca and hindgut. The consequences on the diversity of microbiota in samples have been addressed through 16S metagenomics. Only two samples had a low DNA content resulted in a very low number of reads (caeca of one male from the diet D1at t_120_ and hindgut of one female at t_0_). These two samples have been discarded from the subsequent analyses. For all other samples, the total number of reads obtained for each library ranged from 4 622 to 67 674 (Supplementary Materials Additional file 1).

### 3.1. Digestive Microbial Community Composition

**Figure 2:**
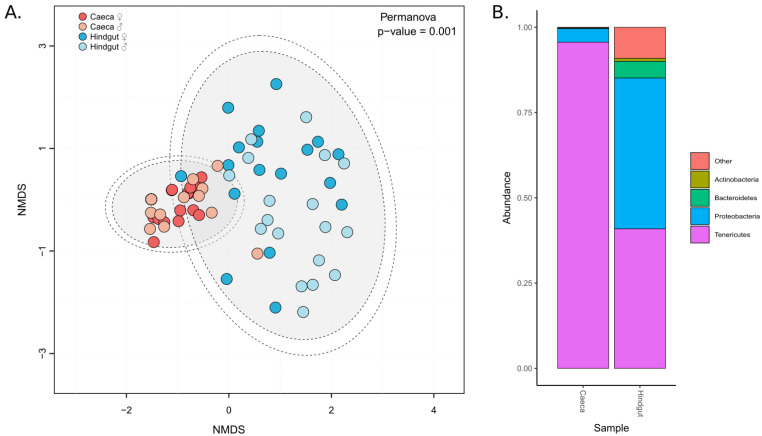
Microbial communities in caeca and hindgut. (**A**) Distribution of microbiota in samples depending on the tissues: caeca in red and hindgut in blue. NMDS were plotted from previously calculated Bray-Curtis dissimilarity matrices, and ellipses were drawn around the centroids of each emerging community (PERMANOVA: F = 17.463, df = 1, *p =* 0.001) at 95% (inner) and 97% (outer) confidence intervals. (**B**) The relative abundance of microbiota composition in caeca and hindgut. All samples from the same tissue were pooled to draw the bar plots.

Bacterial communities hosted by caeca and hindgut were highly different (PERMANOVA: F = 17.463, df = 1, *p =* 0.001), and this regardless the diet conditions (PERMANOVA: F = 1.0052, df = 5, *p =* 0.457), the time point (PERMANOVA: F = 0.355, df = 1, *p =* 0.91) or the sex of the individuals (PERMANOVA: F = 0.861, df = 1, *p =* 0.473) (Figure 2A). Microbiota in caeca was less diversified than the one in hindgut (Supplementary Materials Additional file 2): it was dominated by Tenericutes (representing 95% of the microbiota composition on average) followed by Proteobacteria (3.6% on average), whereas the hindgut hosted Tenericutes in a lower proportion (40.7% on average), Proteobacteria (43.9% on average), Bacteroidetes (4.8% on average), Actinobacteria (1% on average) and 12 other bacterial phyla (Figure 2B).

### 3.2. The Effect of Starvation on Digestive Microbiota

**Figure 3:**
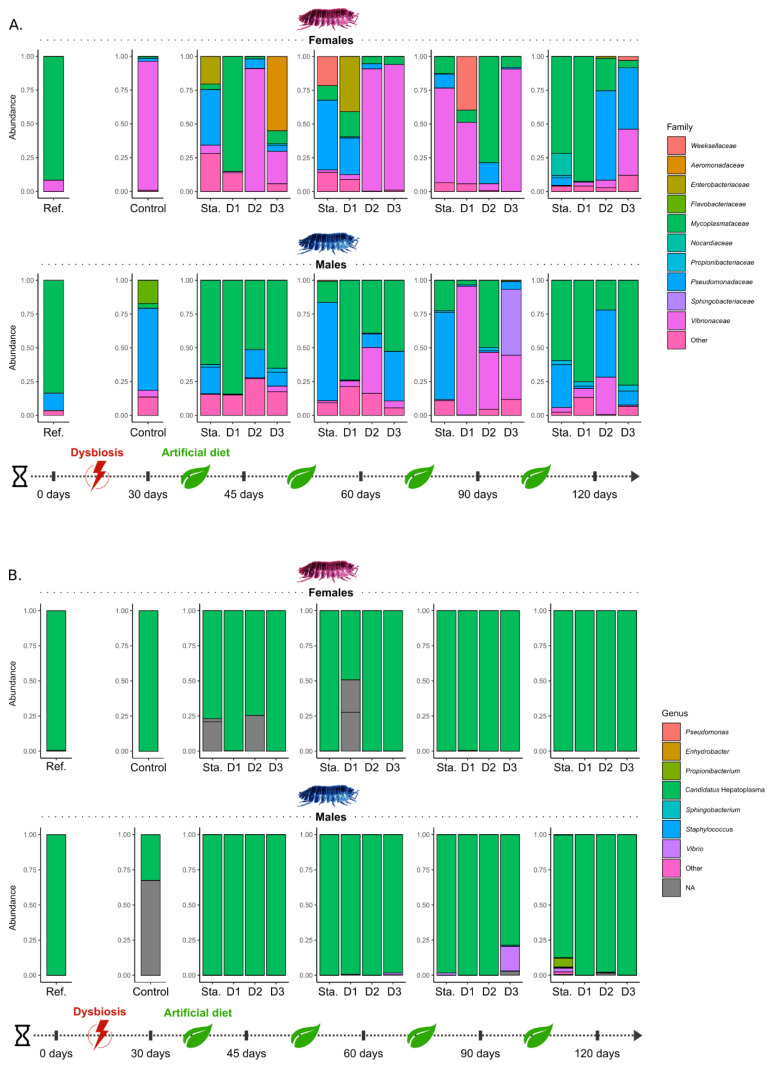
Change of digestive microbiota in samples across time. Bar plots represent relative abundances of microbiota in all samples of males and females in (**A**) hindgut and (**B**) caeca. Microbiota of caeca were represented at the genus level and those of hindguts at the family level for a better understanding.

Significant changes in the composition of the microbiota in the hindgut after the month of starvation (t_30_) were detected by DESeq2 (Figure 3A, Supplementary Materials Additional file 3): although *Mycoplasmataceae* (mostly represented by *Candidatus* Hepatoplasma) dominated in males (83.3%) and females (91.7%) reared in standard conditions (t_0_), they represent less than 4% of the hindgut microbiota in both males and females after one-month starvation (t_30_). This starvation has favored *Vibrionaceae* in females: while they accounted for 8.3% at t_0_, they represent 95.6% of the microbiota at t_30_. In males, *Pseudomonadaceae* increased from 12.9% to 61.1% after this period, as well as *Flavobacteriaceae* (mostly assigned to *Flavobacterium succinicans*) from less than 1% to 17.8%. An increase (log2FoldChange = 21.59, DESeq2 analysis) was also detected in the abundance of bacteria of the *Weeksellaceae* family at the end of the 30-day starvation (Figure 3A, Supplementary Materials Additional file 3). On the contrary, this starvation had no significant impact on microbiota composition in caeca. Microbiota of caeca in females was still largely dominated by the bacteria *Candidatus* Hepatoplasma (*Mycoplasmataceae*, accounting for 99.9% of the bacterial community), as for males even though there was a large proportion of unknown bacteria (68.4%).

The microbiota of the hindgut of males kept in continuous starvation was dominated by *Pseudomonadaceae* and *Mycoplasmataceae* (Figure 3A). In females kept in the same condition, several dominant bacteria were observed in their hindgut, including *Pseudomonadaceae*, *Mycoplasmataceae*, *Weeksellaceae*, *Nocardiaceae,* and *Enterobacteriaceae* (Figure 3A). Compared to the control samples (t_30_), those in continuous starvation had a lower proportion of bacteria of the *Flavobacteriaceae* and *Weeksellaceae* families at almost every time steps (Supplementary Materials Additional file 3). Finally, microbial communities of caeca in both males and females, although they have been maintained in continuous starvation, were still dominated by the bacteria *Candidatus* Hepatoplasma (*Mycoplasmataceae*).

### 3.3. The Effect of Dietary Manipulation on Digestive Microbiota

Differences between microbiota of hindgut in males and females were observed depending on the diets. *Mycoplasmataceae* rapidly recolonized male hindguts in all diets since they represented 51% (D2) to 84.4% (D1) of their microbiota at t_45_ (Figure 3A). In females, microbiota composition depended on the diet and varied greatly over time. In most cases, the majority of sequences belonged to *Vibrionaceae* (D2 at t_45_ and t_60_, D3 at t_60_ and t_90_, D1 at t_90_), followed by *Mycoplasmataceae* (D1 at t_45_, D2 at t_90_ and D1 at t_120_), *Pseudomonadaceae* (D2 and D3 at t_120_), *Enterobacteriaceae* (D1 at t_60_) and *Aeromonadaceae* (D3 at t_45_). Compared to the control samples (t_30_), *Flavobacteriaceae* decreased in all samples, as well as *Weeksellaceae* in almost all samples (Supplementary Materials Additional file 3). Interestingly, *Xanthomonadaceae* increased in the hindgut of individuals fed with the D3 diet as along with those in continuous starvation (Supplementary Materials Additional file 3). Finally, in both males and females, and regardless of the diet, the microbiota is very labile across time. For individuals subjected to the diet D1 (linden leaf and carrots, the standard diet for rearing pill-bugs in our laboratory), the composition of their microbiota (*cf.* reference samples) did not return to its original structure until after 120 days: DESeq2 analysis did not detect significant differences between the reference samples (t_0_) and samples from D1 at t_120_.

Regarding the caeca, there has been almost no significant change in microbiota composition between diets and time (Figure 3B): most of the sequences were assigned to *Candidatus* Hepatoplasma (*Mycoplasmataceae*). However, other bacteria have been identified, such as *Vibrio sp.* (*Vibrionaceae*) representing up to 17.2% of the microbiota of males after 90 days of a diet D3 (cellulosic paper), or *Propionibacterium* (*Propionibacteriaceae*) that represent 6.7% of the microbiota of starved males at t_120_ (Figure 3B).

### 3.4. Changes in Microbiota Potential Functions

**Figure 4:**
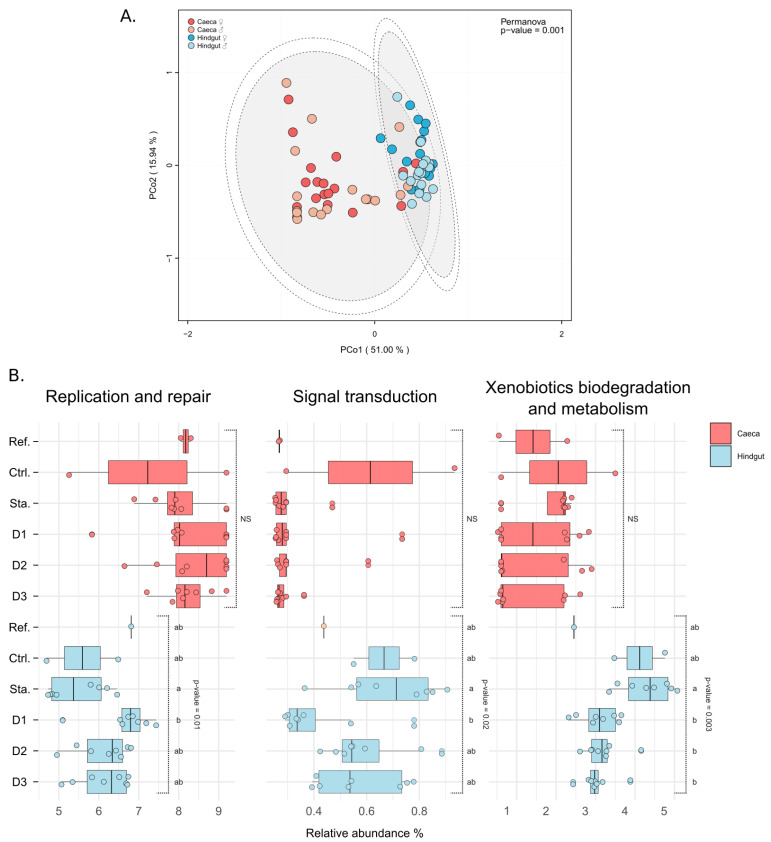
Putative functions in digestive microbiota. (**A**) Distribution of putative functions of microbiota in caeca (in red) and hindgut (in blue). PCoA were plotted from previously calculated Bray–Curtis dissimilarity matrices, and ellipses were drawn around the centroids of each emerging community (PERMANOVA: F = 61.140, df = 1, *p =* 0.001) at 95% (inner) and 97% (outer) confidence intervals. (**B**) Pathways showing significant differences in the abundance of predicted genes as a function of diet in hindgut (in blue): Replication and repair (Kruskal–Wallis: χ2 = 14.7, df = 5, *p =* 0.01), Signal transduction (Kruskal–Wallis: χ2 = 13.0, df = 5, *p =* 0.02) and Xenobiotics biodegradation and metabolism (Kruskal–Wallis: χ2 = 17.8, df = 5, *p =* 0.003).

The abundance of predicted genes was analyzed in microbiota to determine if changes in the composition of microbiota were followed by changes in potential functions. Like taxonomic composition of microbiota, associated predicted functions depended on tissue (PERMANOVA: F = 61.140, df = 1, *p =* 0.001) (Figure 4A). There was no difference in abundance of genes implicated in predicted pathways in caeca between diets, and no variation over time for both the caeca and the hindgut. There are differences between caeca and hindgut in the abundances of genes for 23 of the 31 pathway classes (Supplementary Materials Additional file 4, 5). In the hindgut, only three pathways were varying in gene abundance depending on the diets: Replication and repair (Kruskal–Wallis: χ W2 = 14.7, df = 5, *p =* 0.01), Signal transduction (Kruskal–Wallis: χ2 = 13.0, df = 5, *p =* 0.02) and Xenobiotics biodegradation and metabolism (Kruskal–Wallis: χ2 = 17.8, df = 5, *p =* 0.003) (Figure 4B).

## 4. Discussion

Exposure of *A. vulgare* individuals to starvation and dietary manipulation resulted in significant changes in the composition and functions of their digestive microbiota localized in the hindgut. The microbiota housed in this tissue is highly dependent on the environment and therefore quite labile [20,21,22]. Conversely, the microbiota of caeca was hardly affected by the experiments. Unlike the hindgut, the hepatopancreas is a tissue partially isolated from the environment: particles are filtered at the entrance of these digestive diverticula preventing the passage of many bacteria [29]. In natural populations of *A. vulgare*, the caeca are mainly colonized by two mutually exclusive bacteria: *Candidatus* Hepatoplasma crinochetorum and *Candidatus* Hepatincola [20,47,48,49,50]. These bacteria are dominant and contribute in part to the low diversity of the microbiota in caeca, although some transient bacteria may be present [20]. We demonstrated in this study that the reduced diversity of this microbiota is very stable in both its composition and its functions.

Although sex might be one of the important variables affecting the gut microbiota, we do not show any significant difference between males and females in the present work. In a previous study, we have shown that the main factors affecting the structure of the whole microbiota (including intracellular symbionts) were the origin of the host, the sex, and the presence of *Wolbachia* [20]. However, as feminizing bacteria, *Wolbachia* could act as a confounding factor. It is therefore not surprising that we found no difference in the present study since we used a *Wolbachia*-free lineage of isopods. The same pattern has been recorded in *Ixodes* ticks, where the microbiomes of females are dominated by *Rickettssia* bacteria and are therefore significantly less diverse than those of males [51]. In addition to arthropods, several animal and human studies have shown sex differences in gut microbiota [52]. However, the results are inconsistent and after correcting for confounding factors, such as diet, genotype, and lifestyle, sex only explained a very low proportion of the total variation in the gut microbiota.

The dominant phyla found in the digestive microbiota of the pill-bug were Tenericutes, Proteobacteria, Bacteroidetes, and to a lesser extent Actinobacteria. A similar composition is recorded in the isopod *P. scaber* [53]. These results are in agreement with those obtained in insects where Proteobacteria and Firmicutes are the predominant phyla whereas Clostridiales and Bacteroidales are prevalent in nearly all termites, detritivorous insects [12,54]. In general, insects that specialize in foods high in lignocellulose also have more diverse gut communities but here again, confounding factors such as diet, taxonomic diversity does not give a clear picture [12,54]. After starvation, the structure of the bacterial communities in the hindgut of *A. vulgare* has changed resulting in dysbiosis. While *Candidatus* Hepatoplasma were the major bacteria of the gut microbiota before the starvation, its abundance decreased sharply after this period of stress. Initially described as caeca-associated bacteria [48,49], a recent study revealed its presence in the gut of isopods [20], which was also observed here. Although the role of *Candidatus* Hepatoplasma is unknown in terrestrial isopods, some studies suggest that it may participate in host digestion or provide nutrients [50,55]. Our results suggest that the presence of *Candidatus* Hepatoplasma in the hindgut relies on nutrients available in the gut. Indeed, the abundance of this bacterium drastically decreased in the gut after the month of starvation. This could also explain why it is so abundant in the caeca, the place where nutrients are absorbed and stocked [56,57,58]. In addition, after a return to a standard diet, *Candidatus* Hepatoplasma gradually recolonized the hindgut of several individuals, suggesting its role in food degradation. Nevertheless, it is unlikely to be involved in lignocellulose degradation because no lignocellulose degrading enzyme has been identified in its genome [22,59]. Our analysis of predicted functions showed that genes associated with amino acid metabolism, environmental adaptation, and metabolism of terpenoids and polyketides were more abundant in caeca (largely dominated by *Candidatus* Hepatoplasma), indicating that these bacteria may be involved in one of these pathways.

The decrease in the amount of *Candidatus* Hepatoplasma promoted the development of bacteria from *Vibrionaceae*, *Pseudomonadaceae,* and *Flavobacteriaceae* families in the hindgut. *Vibrionaceae* are well known for their pathogenicity in the hindgut of crustaceans [60,61,62]. They remained abundant throughout the experiments, but their involvement in food digestion is not known. In a recent study, we have identified CAZymes (i.e., Carbohydrate-Active enZYmes [63]) which some are implicated in the lignocellulose degradation in the genome of bacteria of the genus *Vibrio* [22]. Those bacteria could therefore be involved in the digestion of isopods, or simply be opportunistic bacteria that occupy a vacant niche. Conversely, several bacteria from *Pseudomonadaceae* are known to secrete lignocellulose degrading CAZymes in the digestive tract of insects [64,65,66,67]. Surprisingly, they constitute only a small proportion of the microbiota of the digestive tract of pill-bugs fed with the standard diet D1, which was the richest and most nutritious diet. On the contrary, diets of low nutritional value (diet D2 and D3) seem to have favored their presence. The presence of high proportions of lignin and cellulose in these diets may have fostered the growth of those bacteria [68,69]. Finally, bacteria from the *Flavobacteriaceae* family were abundant only in males after one month of starvation. They represented a very small part of the microbiota of both males and females when they started feeding again, only to almost disappear afterward. These bacteria are common in arthropods [70,71,72,73,74,75], and they have already been identified in *A. vulgare* [19,20]. They could be involved in digestive processes as lignocellulose degrading CAZymes have been identified in the *Flavobacteriaceae* of phylum Bacteroidetes in other isopods [21].

Putative functions of the microbiota were inferred by referring to known functions in the closest taxa to those identified. In accordance with the observed bacterial diversity, the functional potential of the microbiota in the caeca would be less diverse than those in the hindgut. Whereas diets appear to not affect the abundance of those genes in caeca, differences in three pathway classes were observed in the hindgut: replication and repair, signal transduction and xenobiotics biodegradation and metabolism appear. Indeed, for these classes, there were differences in gene abundance between starved and dietary individuals. Moreover, differences were more important for individuals subjected to the standard diet compared to those in starvation, thus highlighting the possible impact of starvation on these pathways. Remarkably, no variation in the abundance of the predicted genes was recorded over time in either tissues. This result highlights a potential functional redundancy in both digestive tissues despite the dysbiosis induced by the experiments [76]. This result has also been observed in microbial communities implicated in lignocellulose degradation in isopods: despite very different communities between the different host populations, a functional redundancy has always been observed for lignocellulose degradation [21,22]. This also underlines the stability and resilience of the microbiota of the caeca, a tissue that is more isolated from the external “environment” than the hindgut [29].

## 5. Conclusions

The digestive microbiota of *A. vulgare* is composed of two parts: the one in the caeca, a stable community protected in part from external abiotic and biotic factors, and the one in the hindgut, an unstable community continuously challenged by those factors. However, these differences do not prevent these two communities from interacting, as shown by the presence of the bacteria *Candidatus* Hepatoplasma in the hindgut, probably derived from caeca. The dysbiosis led to a transient state in the hindgut: in a context of relaxed ecological competition, the released niches were colonized by bacteria, most of which were probably opportunistic. The return to selective diets led to new competitions in the hindgut, whose winners depended in part on the type of diet provided. Finally, by altering the structure of the microbiota, dysbiosis and selective diets may have affected the host fitness as shown by the prediction of changes in microbiota functions. It may have affected the host-microbiota interactions by expanding or contracting the ecological niches of the microbiota and its host. The host and its microbiota may mutually modify their ecological niche, thus impacting their respective adaptive value [77]. Such modifications can therefore not only have effects on the holobiont, but also the “eco-holobiont” conceptualization of macroorganisms [78].

## Supplementary Materials

The following are available online at https://www.mdpi.com/2076-2607/9/1/148/s1, Additional file 1: Metrics of the metagenomics samples. (XLSX); Additional file 2: Diversity indexes (Shannon’s index and Pielou’s evenness) of the microbiota. (PDF); Additional file 3: Differential relative abundance comparisons of microbiota between control samples (t_30_) and samples from the different conditions using DESeq2: A = significantly differentially abundant bacterial families between Reference and Control conditions; B = significantly *differentially abundant* bacterial families between Diet conditions over time (PDF); Additional file 4: Predicted pathway classes and associated gene abundances. (XLSX); Additional file 5: Differentially abundant genes in pathways classes between caeca and hindgut.
