# Supplementary material for: Effects of Dysbiosis and Dietary Manipulation on the Digestive Microbiota of a Detritivorous Arthropod
Source: Microorganisms. 2021 Jan 11;9(1):148. doi: 10.3390/microorganisms9010148 (PMC7826753; doi:10.3390/microorganisms9010148)
Supplement: Supplementary file 1 [file microorganisms-09-00148-s001.zip › Additionnal_file_3.pdf]

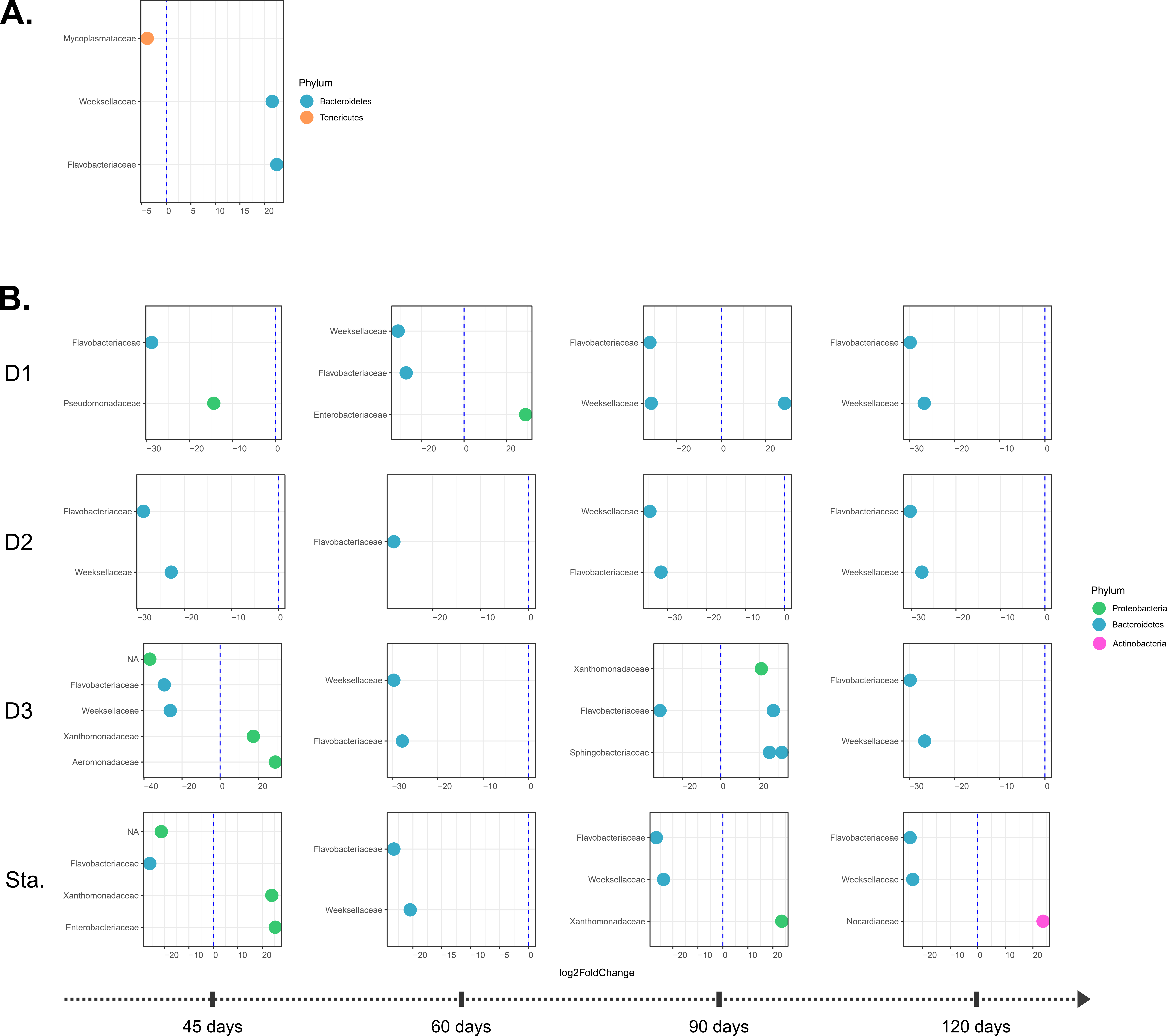

A.
Mycoplasmataceae
Weeksellaceae
Flavobacteriaceae
−5 0 5 10 15 20
Phylum
Bacteroidetes
Tenericutes
B.
D1
D2
D3
Sta.
Flavobacteriaceae
Pseudomonadaceae
Weeksellaceae
Enterobacteriaceae
NA
Xanthomonadaceae
Aeromonadaceae
Sphingobacteriaceae
Nocardiaceae
Phylum
Proteobacteria
Bacteroidetes
Actinobacteria
log2FoldChange
45 days
60 days
90 days
120 days
