# Supplementary figures and images for: Effects of Dysbiosis and Dietary Manipulation on the Digestive Microbiota of a Detritivorous Arthropod
Source: Microorganisms. 2021 Jan 11;9(1):148. doi: 10.3390/microorganisms9010148 (PMC7826753; doi:10.3390/microorganisms9010148)

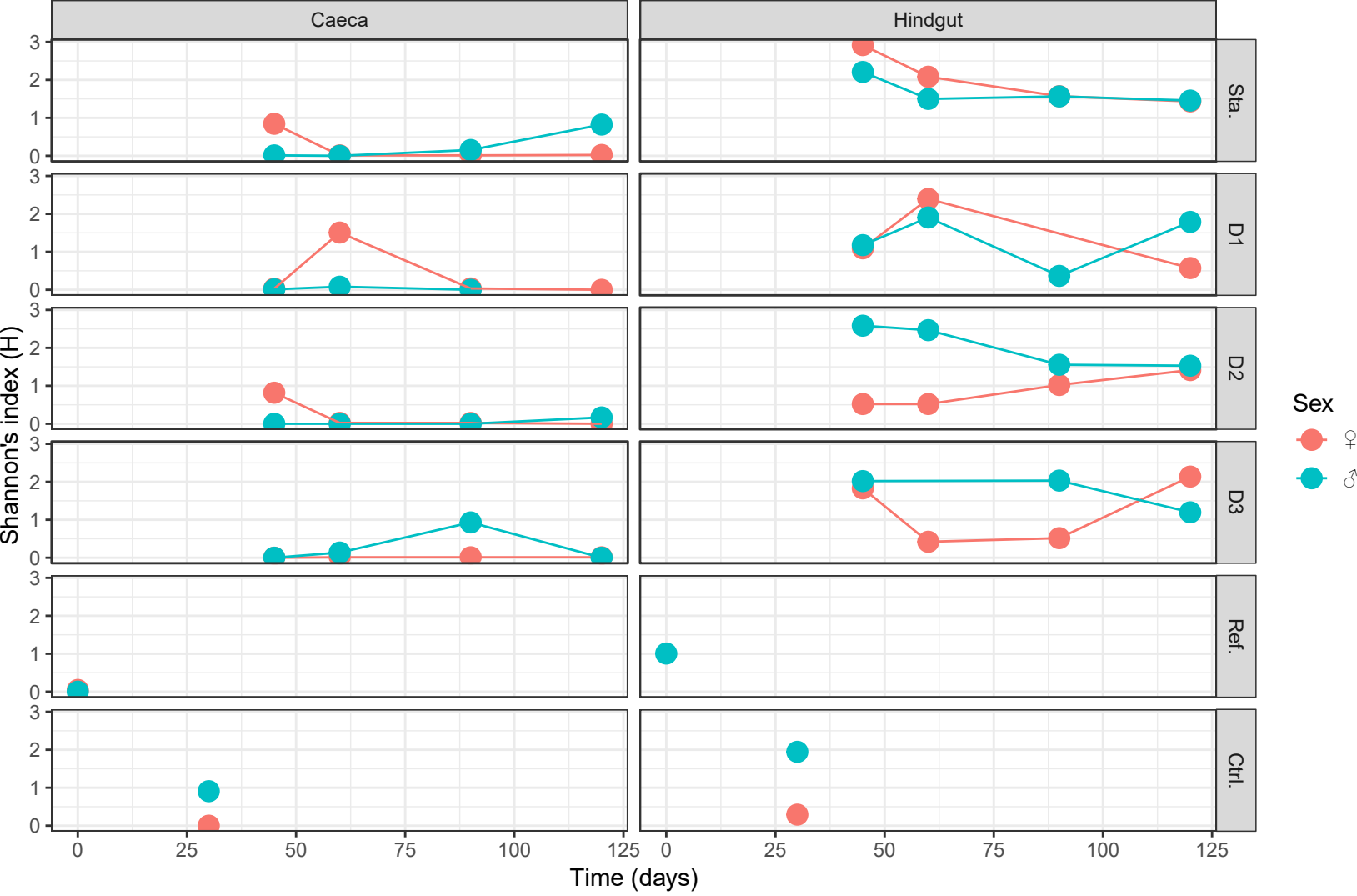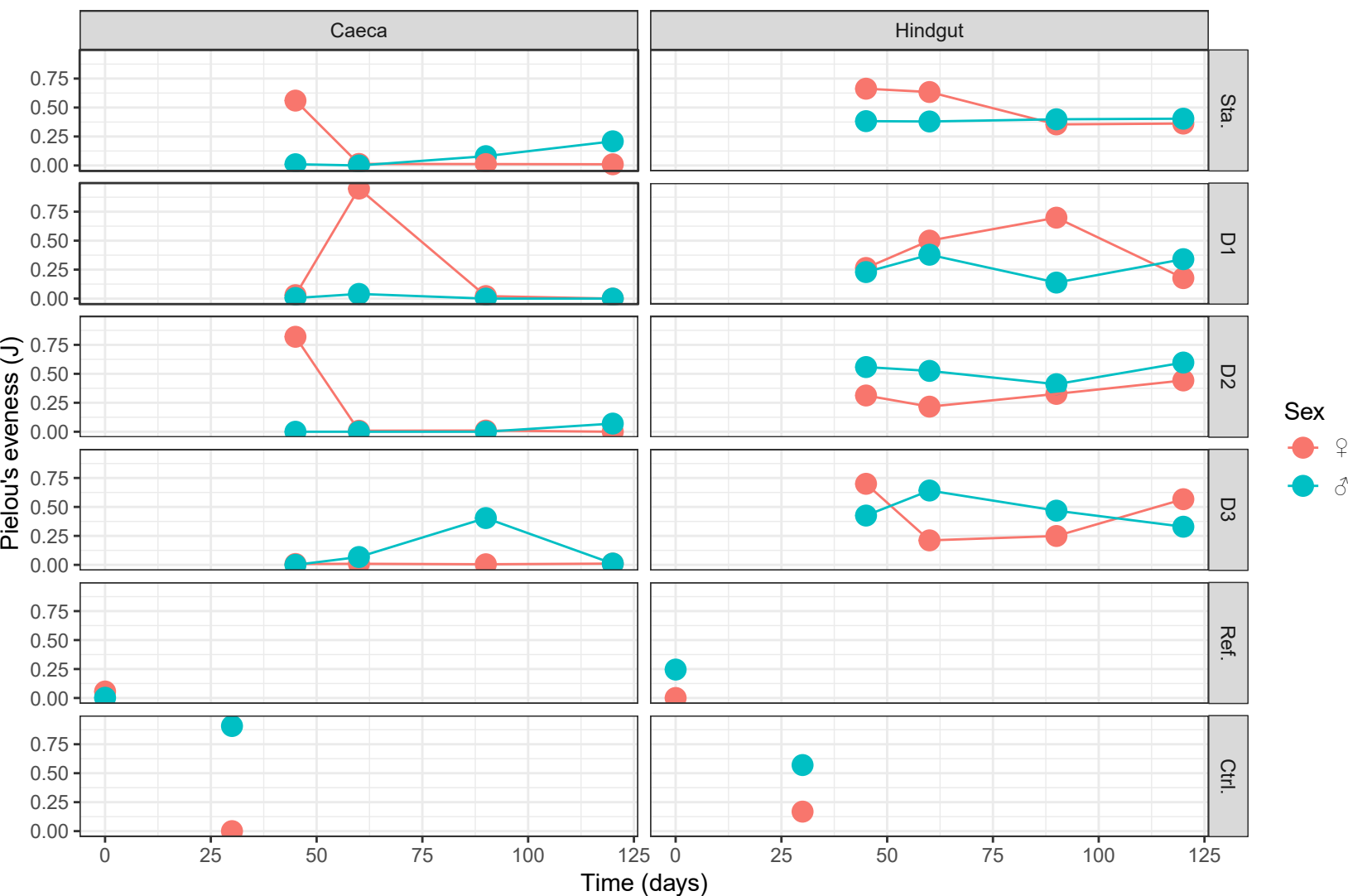

Supplement: Supplementary file 1 [file microorganisms-09-00148-s001.zip › Additionnal_file_2.pdf]

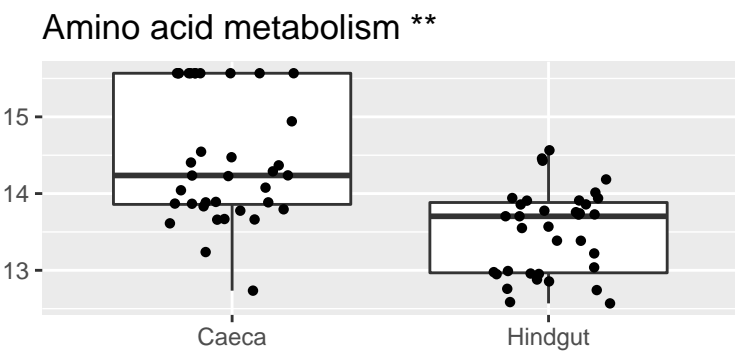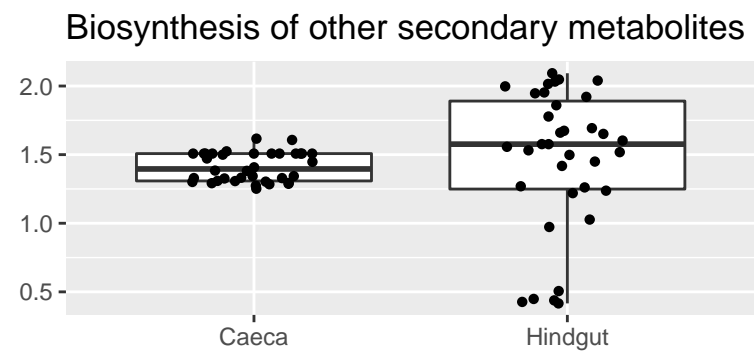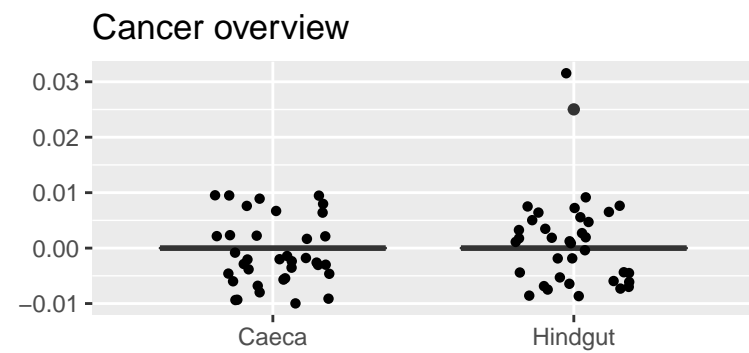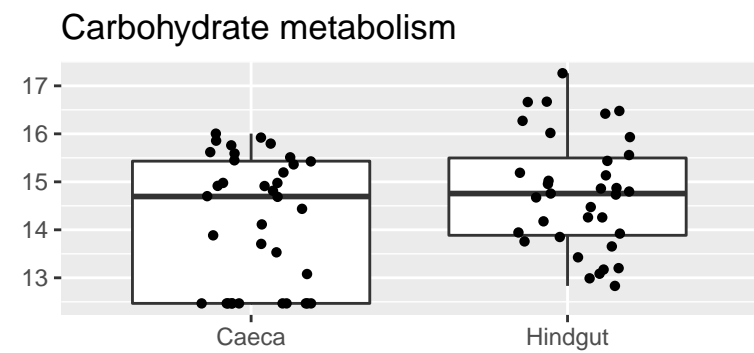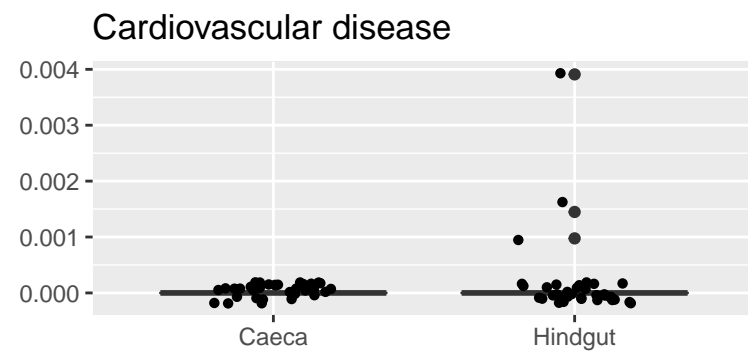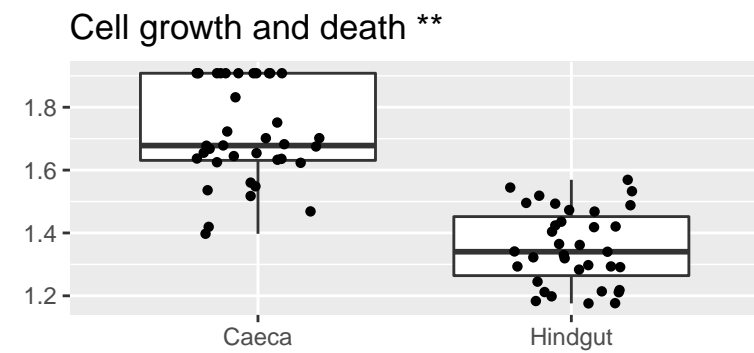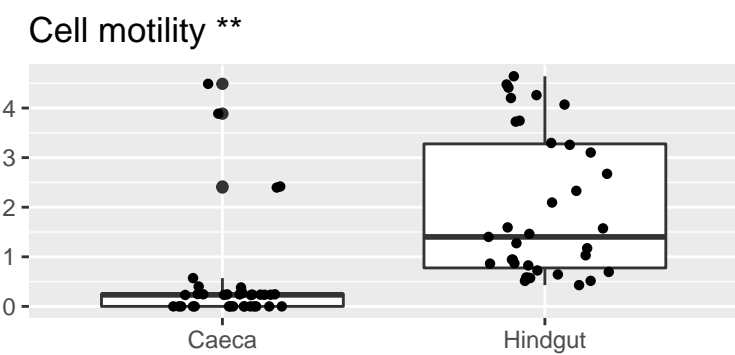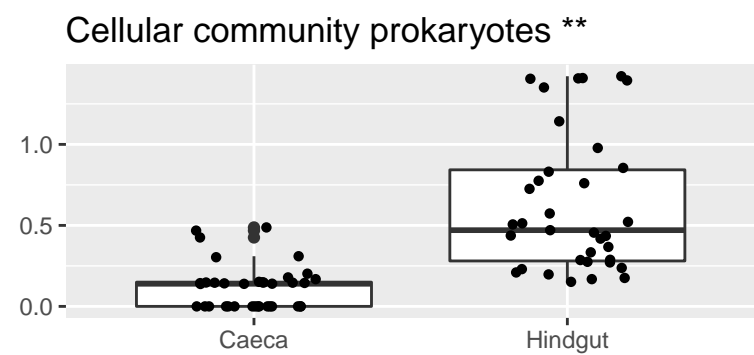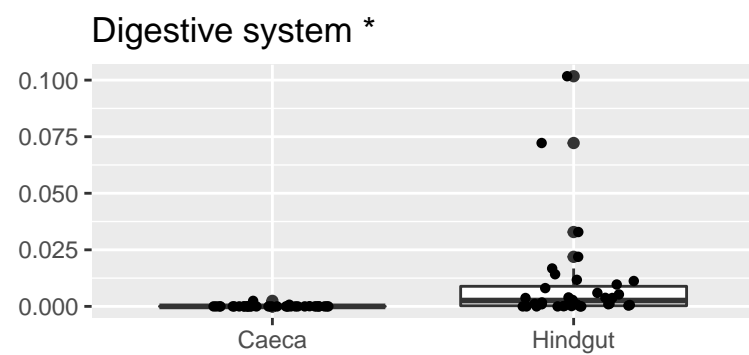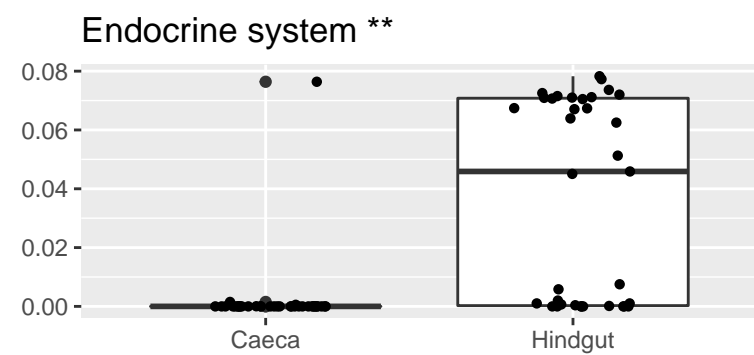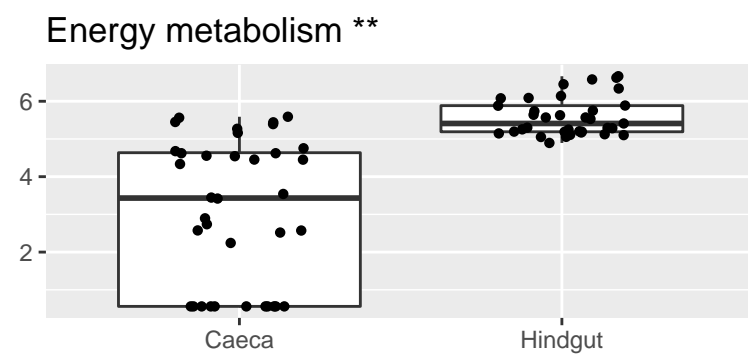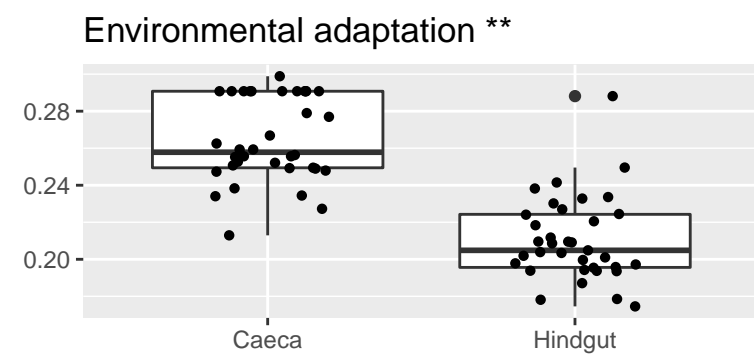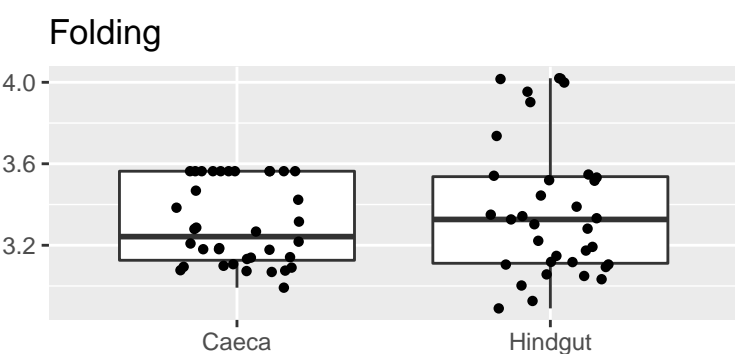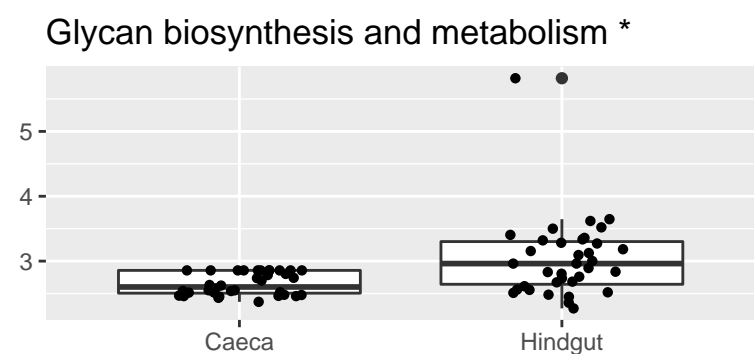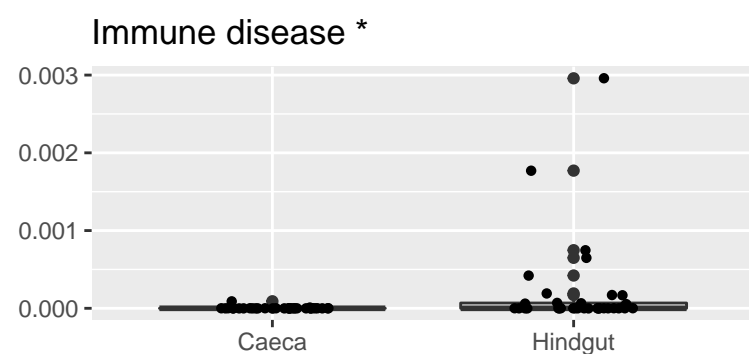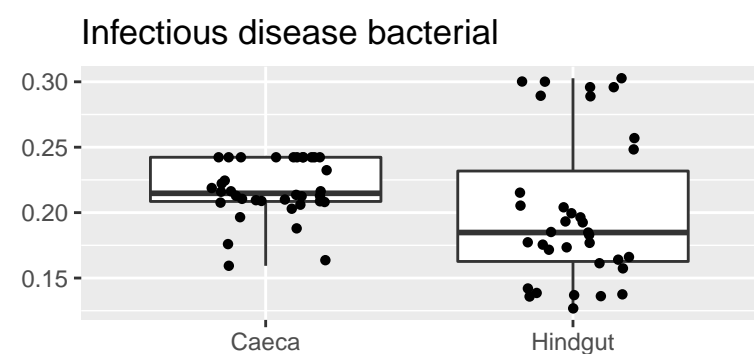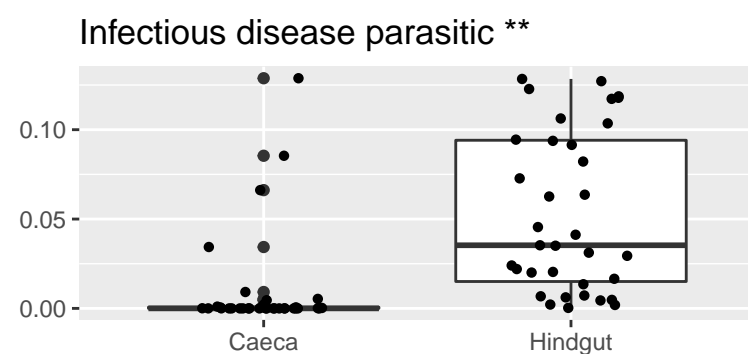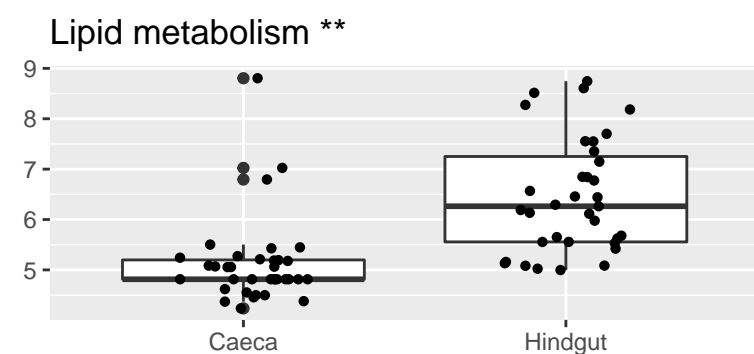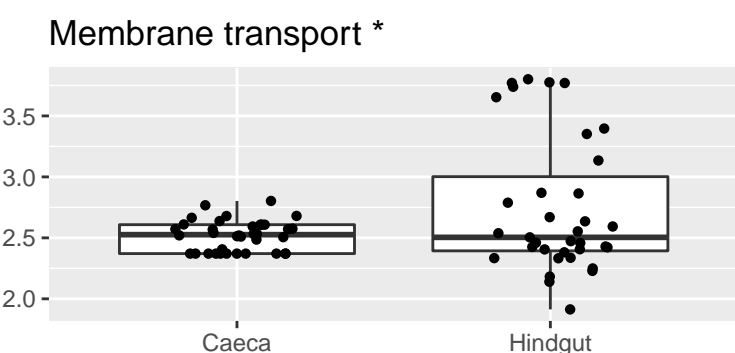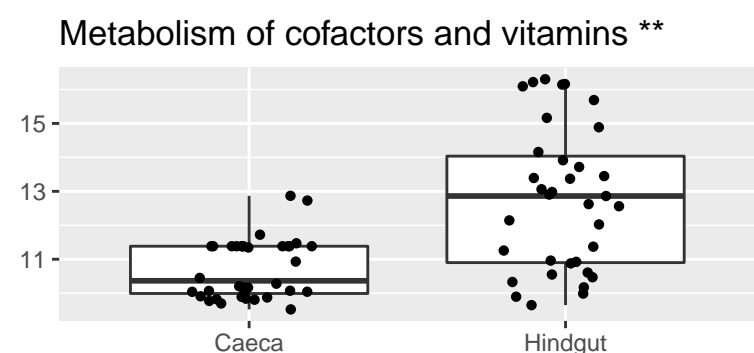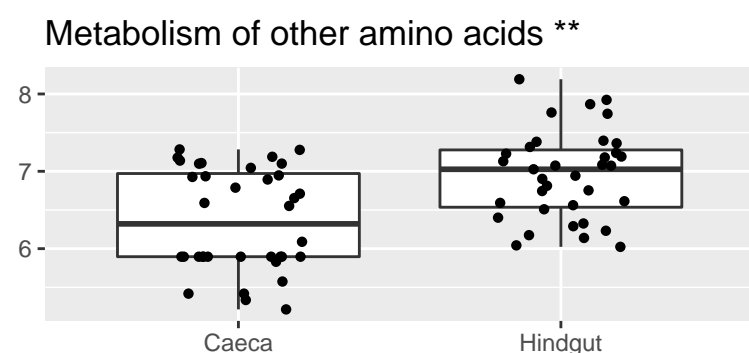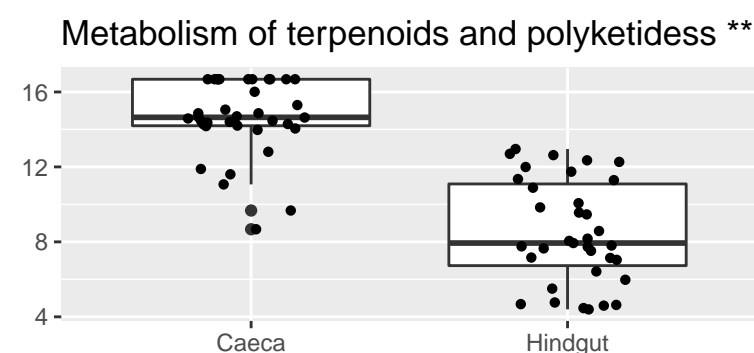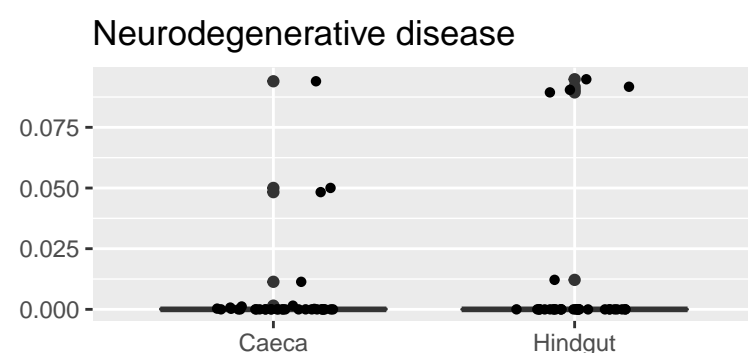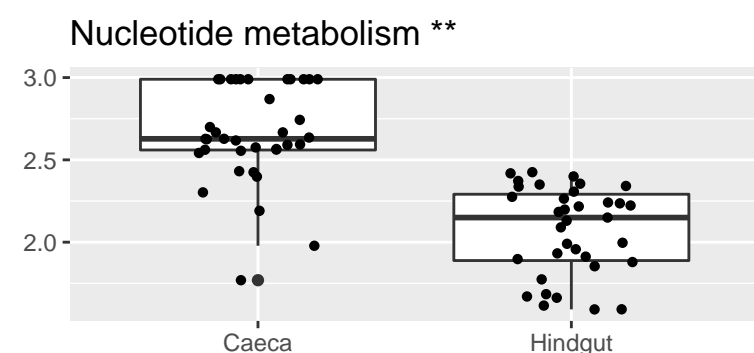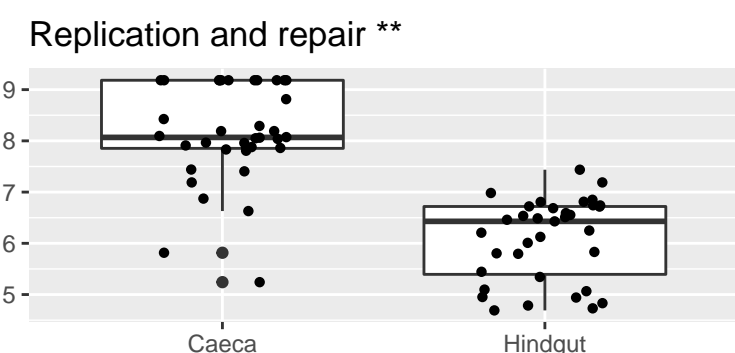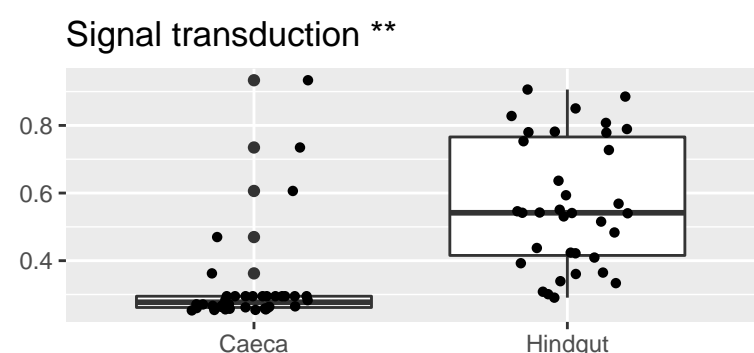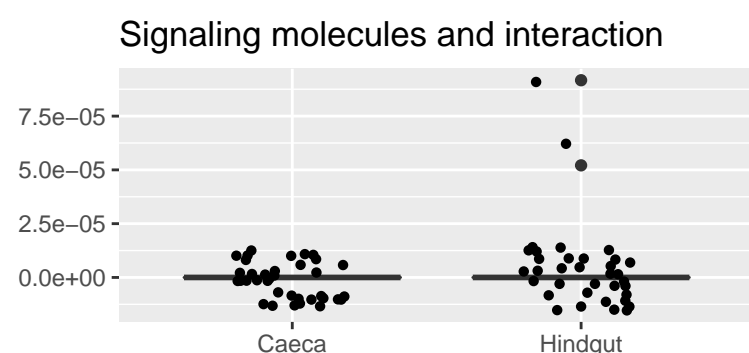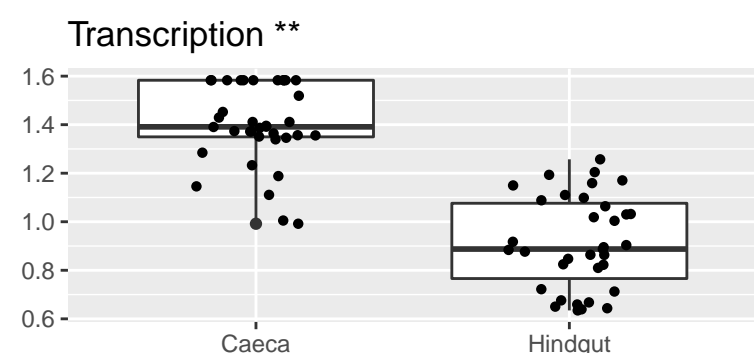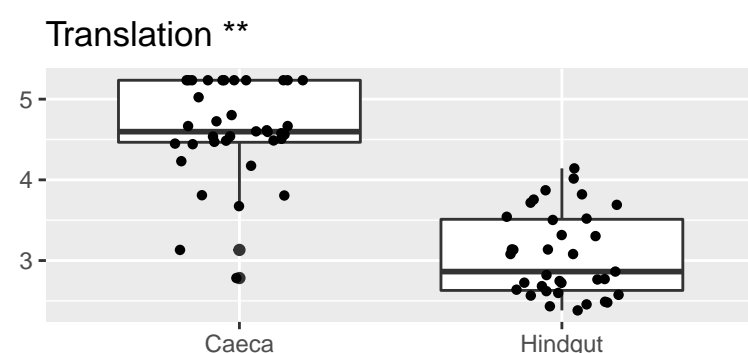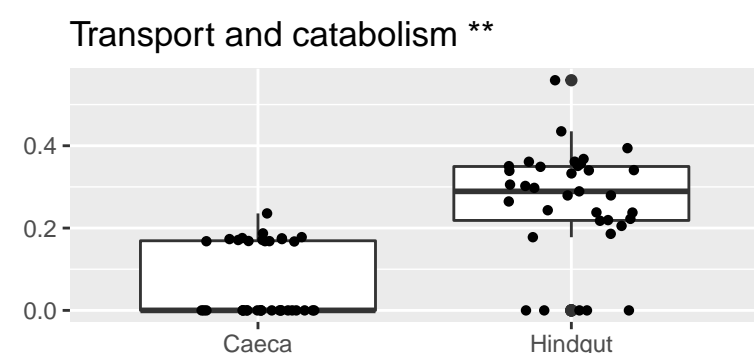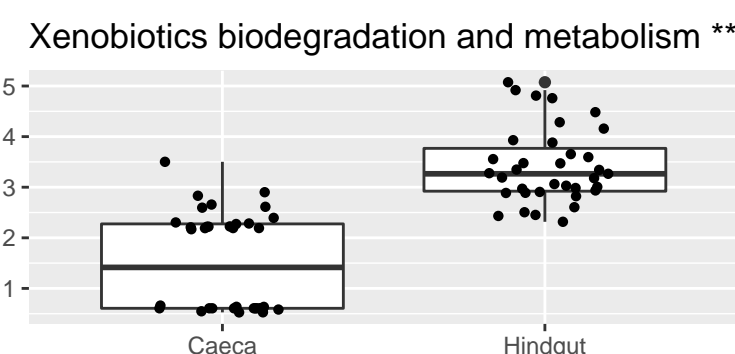

Supplement: Supplementary file 1 [file microorganisms-09-00148-s001.zip › Additionnal_file_5.pdf]
